# Genetic fine-mapping of the Iowan *SNCA* gene triplication in a patient with Parkinson’s disease

**DOI:** 10.1038/s41531-018-0054-4

**Published:** 2018-06-15

**Authors:** Faria Zafar, Ruksana Azhu Valappil, Sam Kim, Krisztina K. Johansen, Anne Lynn S. Chang, James W. Tetrud, Peggy S. Eis, Eli Hatchwell, J. William Langston, Dennis W. Dickson, Birgitt Schüle

**Affiliations:** 1 Parkinson’s Institute and Clinical Center, Sunnyvale, CA USA; 2 Department of Neurology, Akershus University Hospital, Lorenskog, Norway; 3 Department of Dermatology, Stanford University School of Medicine, Stanford, CA USA; 4 Stanford Neuroscience Health Center, Stanford School of Medicine, Stanford, CA USA; 5 Population Bio, Inc, New York, NY USA; 6 Neuropathology Laboratory, Mayo Clinic, Jacksonville, FL USA

## Abstract

The “Iowa kindred,” a large Iowan family with autosomal-dominant Parkinson’s disease, has been followed clinically since the 1920s at the Mayo Clinic. In 2003, the genetic cause was determined to be a 1.7 Mb triplication of the alpha-synuclein genomic locus. Affected individuals present with an early-onset, severe parkinsonism-dementia syndrome. Here, we present a descendant of the Iowa kindred with novel, disease-associated non-motor findings of reduced heart rate variability, complete anosmia, and a rare skin condition called colloid milium. At autopsy, key neuropathological findings were compatible with diffuse Lewy body disease. Using high-resolution comparative genomic hybridization (CGH) array analysis to fine-map the genomic breakpoints, we observed two independent recombination events of the *SNCA* locus that resulted in a genomic triplication of twelve genes, including *SNCA*, and the disruption of two genes, *HERC6* and *CCSER1*, at the genomic breakpoints. In conclusion, we provide further evidence that the mere two-fold overexpression of alpha-synuclein leads to a fulminant alpha-synucleinopathy with rapid progression and severe clinical and neuropathological features.

## Introduction

The Iowa kindred was initially described by Spellman in 1962 as a family with severe parkinsonism of autosomal-dominant inheritance.^1^ The family is of English and German descent with early age at onset (average 34 years, range 20–48 years). Disease progression is rapid with dementia and death occurring within 2–12 years after onset of symptoms. Neuropathology revealed severe degeneration of the substantia nigra in this kindred, widespread subcortical and cortical Lewy bodies, vacuolation of the cortex, nerve cell loss, and gliosis in the hippocampus. Neuritic pathology in cortical areas was detected by alpha-synuclein staining, which exceeded the magnitude of the most severe cases of dementia with Lewy bodies.^2–5^

In 2003, the underlying genetic cause was determined by Singleton and collaborators to be a triplication of the *SNCA* genomic locus on chromosome 4q21.^6^ Using quantitative PCR, the size of the triplication was initially narrowed down to a range between 1.61 and 2.04 Mb, containing 17 genes. A triplication of the *SNCA* gene on one allele (in addition to one copy of alpha-synuclein on the wildtype allele) causes two-fold up-regulation of the alpha-synuclein protein in the brain. This discovery demonstrated that a mere overexpression of wild-type alpha-synuclein could lead to a neurodegenerative condition very similar to Parkinson’s disease (PD). Two genetic screens in autosomal-dominant parkinsonism with dementia reported a frequency of duplications and triplications of ~1.5%.^7,8^ In a follow-up study, breakpoints for different families with chromosomal gains (duplications and triplications) were analyzed using SNP array technology and the Iowa kindred *SNCA* triplication was estimated to be ~1.7 Mb.^9^

Herein, we describe novel clinical, neuropathological and refined molecular genetic findings in a descendant from the Iowa kindred. We report non-motor findings such as reduced heart rate variability (HRV) and complete anosmia, and findings of a rare skin condition, colloid milium, which has not been previously reported in association with PD. Using high-resolution array comparative genomic hybridization (CGH) for copy number variant (CNV) analysis, we found that the *SNCA* triplication breakpoints disrupt two genes (*HERC6* and *CCSER1*).

## Results

### Clinical description of presented case

Our patient presented at age 41 with rapidly progressive parkinsonism. The disease started with excessive fatigue, resting tremor in left arm, and a change in speech. MRI of the brain was unremarkable. He was treated with levodopa and his symptoms improved substantially. A year after levodopa treatment he experienced motor fluctuations. By age 43, symptoms progressed and his mental function was deteriorating quickly. At age 44, he showed typical motor complications with wearing off phenomenon, gait freezing, and peak-dose dyskinesias. He complained of bladder urgency, occasional constipation, and orthostatic hypotension. He reported vivid dreams and was “thrashing around” during his sleep, consistent with REM sleep behavior disorder.

A neurological examination at age 41 revealed moderate resting tremor and slight action tremor in his left hand and moderate rigidity bilaterally. Rapid sequential movements were moderately slow bilaterally. His posture was moderately stooped. His gait was normal with only reduced arm swing bilaterally and the patient recovered unaided from the pull test. His MOCA score was found to have dropped from 30/30 at age 41 to 8/30 at age 45. Olfactory function was tested at age 45 using the 40-item UPSIT. His score of 11/40 represents total anosmia (below 5th percentile) (Supplemental Fig. 1). We tested HRV and found extremely reduced HRV in a 5 min resting EKG. The HRV parameters measured were standard deviation of RR intervals (SDNN), percentage of consecutive RR intervals differing by more than 50 msec (pNN50), RR triangular index (RRTRI), width of Poincare plot SD1, low frequency (LF) normalized and low frequency/high frequency (LF/HF) ratio. The *SNCA* triplication patient had in all parameters measured lower values than any of the idiopathic PD patients tested (Supplemental Fig. 2) representing severe autonomic dysfunction. The Epworth Sleepiness Scale determined his level of daytime sleepiness to be 9/24, within the high normal range of 0-10. He demonstrated low color discrimination with a total error score of 124 on the Farnsworth-Munsell 100 Hue test (mean 68 for normal age group 40-49 yrs^10^).

His condition deteriorated rapidly prompting full-time care in a nursing home after only 6 years following diagnosis at age 47 and death at age 50.

Past medical history prior to onset of PD was unremarkable aside from a rare skin condition called colloid milium (Supplemental Figure 3), manifested by multiple dense dome-shaped pink and yellow papules on the dorsum of both hands and up to the elbows. This condition is thought to be caused by exposure to the sun, petroleum products, or skin bleaching creams containing hydroquinone.^11,12^

The patient is a descendent of the Iowa kindred^4–6^ and a neuropathological case report has been published on his mother. Age at onset in his mother was at 45 years with a similar rapidly progressive course including dementia.^3^

### Neuropathology

**Fig. 1:**
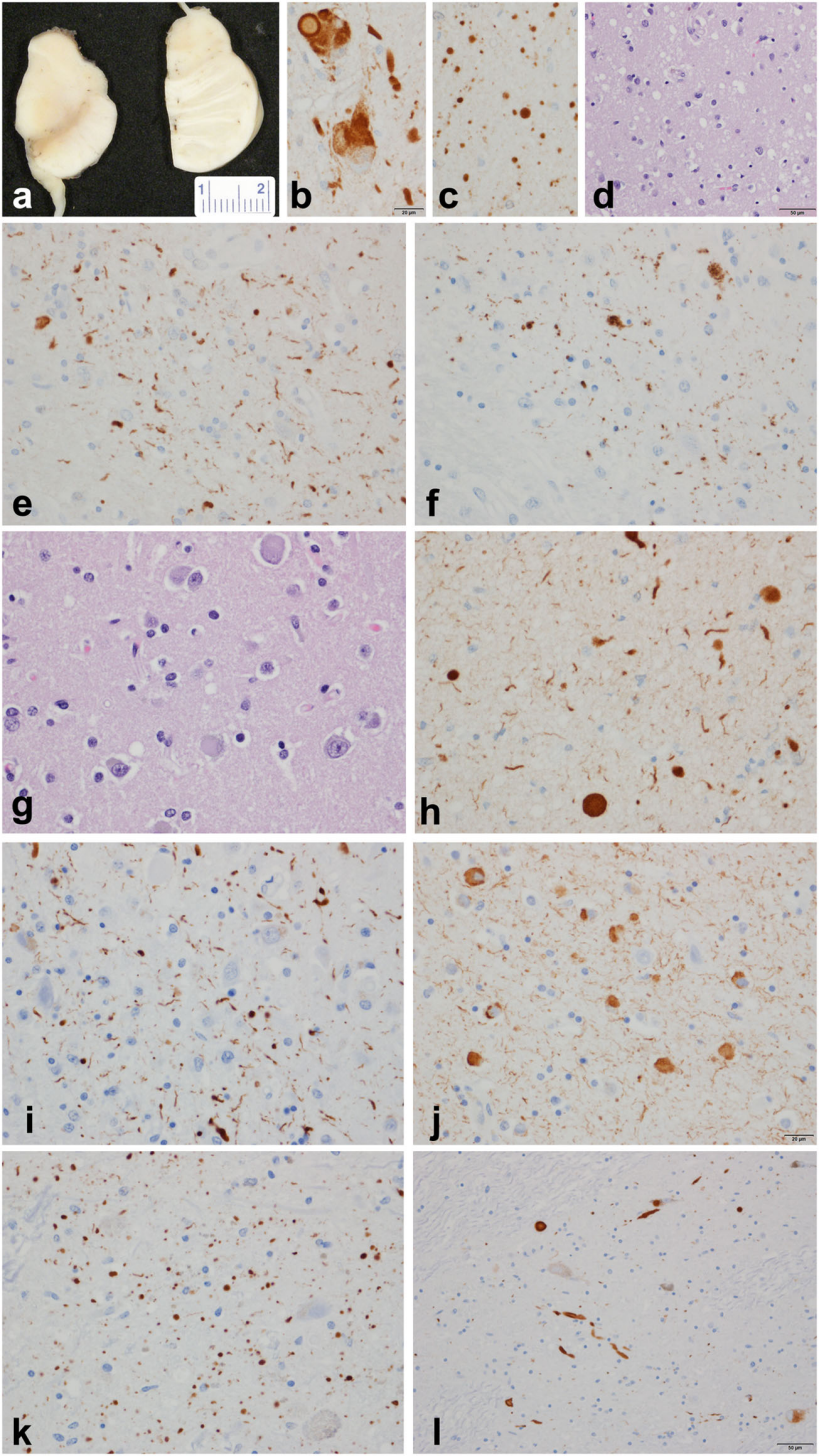
*SNCA* genomic triplication neuropathology. **a** Gross pathology of midbrain and pons with loss of neuromelanin pigment in both; **b** alpha-synuclein immunohistochemistry of locus ceruleus with Lewy bodies and bizarre neuronal inclusions; **c** Numerous Lewy dots in ventral tegmental region of midbrain; **d** spongiform change in neocortex in temporal and limbic lobes; **e** CA2 sector of hippocampus with Lewy neurites; **f** CA2 sector of hippocampus with tau in subset of Lewy neurites; **g**, **h** cortical Lewy bodies in temporal neocortex; cortical Lewy bodies and Lewy neurites in temporal neocortex; **i** hippocampal CA2 neurites; **j** amygdala Lewy bodies and neurites; **k** ventral tegmental area Lewy neurites (“Lewy dots”); **l** substantia nigra pars compacta Lewy bodies.alpha-synuclein immunohistochemistry (**b**, **c**, **e**, **h**), phospho alpha-synuclein (**i**, **j**, **k**, **l**), tau immunohistochemistry (**f**), hematoxylin and eosin stain (**d**, **g**). Bar in **b** = 20 μm (applies to **c**, **e**, **f**, **g**, **h**, **i**, **j**, and **k**); bar in **d** and **l** = 50 μm; measure bar in **a** is in mm

Alpha-synuclein immunoreactive cortical Lewy bodies and Lewy neurites were most numerous in limbic and paralimbic cortices, parahippocampal cortex, insular cortex, cingulate gyrus, and in the anterior olfactory nucleus of the olfactory bulb. Additionally, there were Lewy bodies, Lewy neurites and glial inclusions in the spinal cord gray matter at the cervicomedullary junction (Fig. 1).

The basal nucleus of Meynert showed severe neuronal depopulation. Lewy bodies and Lewy neurites were numerous in the basal forebrain and hypothalamus. The amygdala had neuronal loss and marked gliosis and mild spongiform change with many Lewy neurites (curvilinear and dot-like) and Lewy bodies, especially in the cortical transition zone (25–45 per 20 × field) (Supplemental Table 1). α-synuclein immunohistochemistry showed many Lewy neurites, scattered glial inclusions and many spheroids in the globus pallidus. The putamen had numerous dot-like and curvilinear neurites and many cortical-type Lewy bodies as well as glial inclusions. The thalamus and subthalamic nucleus were unremarkable, but there were spare Lewy bodies and Lewy neurites in the anterior and medial nuclei.

The substantia nigra had severe neuronal loss with only sparse extraneuronal neuromelanin. There were Lewy bodies in residual neurons and many Lewy neurites, Lewy dots, and glial inclusions. Lewy bodies were present in the raphe nuclei and periaqueductal gray. The locus ceruleus had neuronal loss and gliosis with Lewy bodies. There were many Lewy bodies and neurites in the mesopontine tegmentum. A few Lewy neurites were even detected in the pontine base. The medulla was remarkable for intraneuritic Lewy bodies, Lewy neurites and neuronal loss in the dorsal motor nucleus of the vagus. There were many Lewy bodies and Lewy neurites in the medullary tegmentum. A few glial inclusions were noted in the inferior olivary nucleus. The cerebellum showed well preserved Purkinje and internal granular cell layers. There were rare glial inclusions in the cerebellar white matter with α-synuclein immunohistochemistry. The pituitary was histologically unremarkable. There was no TDP-43 pathology. Medial temporal lobe tau pathology was detected consistent with early argyrophilic grain disease.^13^

### SNCA duplication/triplication breakpoints

**Fig. 2:**
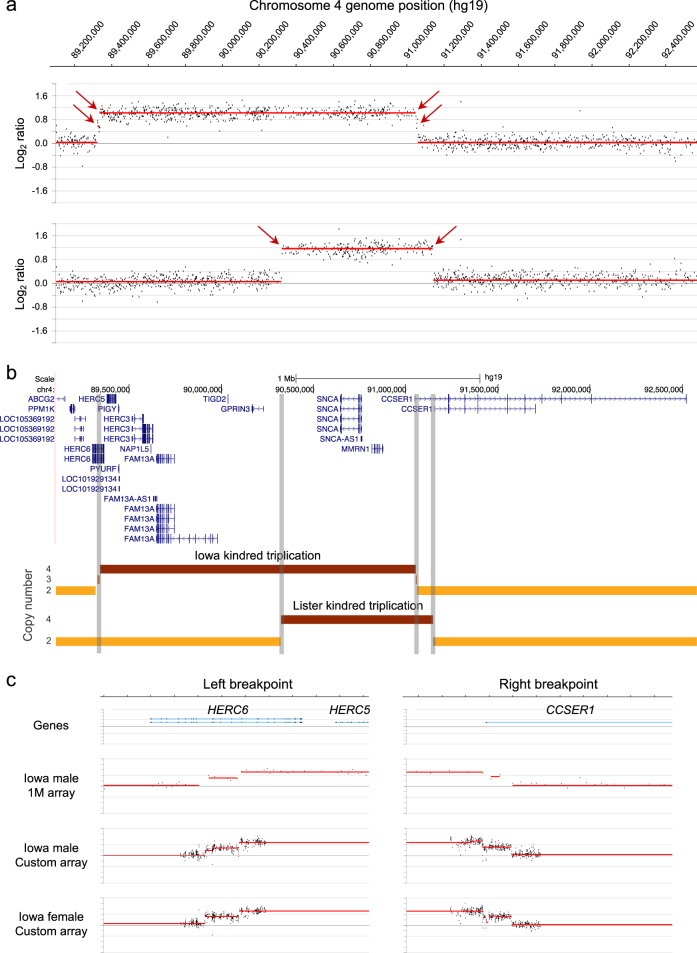
Chromosomal breakpoint map of *SNCA* triplications. **a** Array CGH data for the Iowa kindred case (top data track) and a patient from the Lister kindred (bottom data track). The Iowa triplicated region is 1.7 Mb and the Lister triplicated region is 0.8 Mb. Duplication and triplication breakpoints determined by the algorithm DNAcopy are indicated by red arrows. **b** Genome browser (UCSC, hg19) view of RefSeq genes located within and flanking the *SNCA* triplications. Copy numbers of 2, 3, and 4 are denoted by light, medium, and dark orange line segments for the *SNCA* triplication patients shown in panel (**a**). Vertical gray-shaded bars demarcate the breakpoints in the gene track. **c** Array CGH fine-mapping of Iowa kindred breakpoints: top data track is a zoomed view of the 1 M probe array data (panel **a**) on the Iowa male case report, middle data track is the Iowa male using a higher resolution custom CGH array, bottom data track is an Iowa female on the custom CGH array. See supplemental table 2 for genome coordinates. A Log2 ratio value of 0 corresponds to no change in copy number relative to a reference genome (see Methods), whereas duplicated and triplicated chromosomal regions have Log2 ratio values of ~0.6 and ~1.0, respectively

To better understand the genomic structure and size of the *SNCA* triplication, we performed high resolution CNV analysis (1 M CGH array) to refine the breakpoints of the *SNCA* triplication (Fig. 2). For comparison, we also performed CNV analysis on another *SNCA* triplication patient (Lister kindred, Swedish–American descent),^14,15^ whose right breakpoint closely maps to the one found in the Iowa kindred.^9^ Evidence of a common founder is unlikely based on chromosome 4q21 microsatellite analysis for markers D4S3474, D4S3479, and D4S3476. The Iowa kindred has allele lengths of 185/197/205, 163/165/167, and 316/332/334,^9^ whereas the Lister kindred carries alleles 199/203, 163/167/169, and 332/334.^9,15^ The results from the 1 M CGH array (Fig. 2) clearly delineate the regions of triplication in these two cases. Interestingly, small regions of a duplication were also detected in our patient with a high-density 60 K custom CGH array (Fig. 2c), suggesting two independent mutational events. We find in our higher resolution CNV analysis that both the left and right breakpoints disrupt genes, *HERC6* (left breakpoint) and *CCSER1* (right breakpoint) (Fig. 2c and Supplemental Table 2). In silico analysis of the breakpoint regions revealed several repetitive elements, a LINE L2a and L2c as well as a LTR26E element, however, we did not find overt homology that would explain the rearrangement events (Supplemental Figure 5).

We confirmed the disruption of both genes by mRNA expression analysis and found decreased expression for both *HERC6* and *CCSER1* compared to *SNCA* and *MMRN1* in induced pluripotent stem cells (iPSCs) from our patient differentiated into dopaminergic neurons compared to cells from a sibling control (Supplemental Figure 6). We tested expression for SNCA, MMRN1, CCSER1, as well as three HERC genes in the triplicated region, HERC3, HERC5, and HERC6. All genes in the *SNCA* triplication region showed an increase in mRNA expression between 2–3.5 fold compared to iPSCs from a sibling control (Supplemental Figure 6A). When we differentiated the iPSCs into a mixed population of dopaminergic neurons with ~25–35% of neurons expressing tyrosine hydroxylase (TH), the rate limiting enzyme for dopamine synthesis, we found that the *SNCA* and *HERC3* gene expression were 2-fold increased, *MMRN1* expression was ~6-fold elevated, and *HERC5* mRNA expression was markedly reduced compared to the sibling control possibly due to disrupted regulatory genomic elements. Also as shown in previous work from our group, the expression of TH was significantly reduced as compared to a healthy sibling control.^16^

## Discussion

Non-motor symptoms are becoming increasingly important and critical for early diagnosis of PD. In recent studies, sense of smell^17–21^ and cardiac autonomic denervation, which can be measured as reduction of HRV^22,23^ or myocardial scintigraphy (MIBG),^24,25^ have been used as early clinical indicators of PD.

In this report, we show that our patient with the *SNCA* gene triplication scores the lowest for both sense of smell on the UPSIT and has the lowest HRV values overall in a group of sporadic, typical PD. It is noteworthy that our patient presented here is also the youngest subject studied. Our patient and other published cases^8,15,17,26^ hint that patients with *SNCA* genomic triplications exhibit impaired olfaction more severe than other *SNCA* variants (duplications and single base mutations), possibly due to a dose effect of alpha-synuclein protein.

The other interesting clinical finding in our patient is the presentation of skin lesions, called colloid milium, a rare skin condition that has not been reported in conjunction with PD. Only one other report links this skin disorder to a genetic disease beta-thalassemia.^27^ The cause of colloid milium is unknown, however, high exposure to sun, petrol substances, and dermal bleaching creams are thought to have an impact on this condition.^11,12^

The neuropathological findings in our case are consistent with diffuse Lewy body disease with typical distribution and density of cortical Lewy bodies and severe CA2/3 neuritic pathology in addition to the severe neuronal loss in the ventrolateral cell group of the substantia nigra. Similar neuropathology has been described in other affected individuals of the Iowa kindred including the mother of our patient.^3^

Refined genomic breakpoint analysis of the DNA in our patient with the *SNCA* genomic triplication revealed that there are small regions at the left and right breakpoint of a duplication (Fig. 2c), suggesting two independent mutational events. A dynamic *SNCA* genomic multiplication has been reported in the Lister family complex^15^ where a patient with an *SNCA* duplication (Branch J) and a patient with a triplication (Branch I) presented with shared haplotype of the *SNCA*/*MMRN1* region and based on an extended pedigree published in 1949, a common ancestor could be determined. However, we did not detect any evidence of a duplication in the subject of the Lister kindred (Fig. 2). Additional published duplication/triplication rearrangements are shown in Supplemental Figure 4 to illustrate the variability and size of CNVs of this region.^28^

Furthermore, we narrowed down the left and right genomic breakpoints of the triplication and found that the *HERC6* and *CCSER1* genes were disrupted. This is in contrast to a lower resolution CNV analysis of this patient that mapped the breakpoints to a region between *HERC6* and *HERC5* and an adjacent region outside of the *CCSER1* gene.^9^ The impact of these novel findings is that there is now evidence that the Iowa and Lister kindreds both have a triplication breakpoint (right) that disrupts *CCSER1*, which may account for PD or non-PD phenotypic similarities in these patients/families. In contrast, the *HERC6*-disrupting breakpoint we found in our Iowa kindred case may account for phenotypic differences between these two kindreds.

Based on these new genetic findings in our patient, it is interesting to discuss if one or both gene disruptions are contributing to the patient’s clinical phenotypes reported herein. The *CCSER1* gene (Coiled-Coil Serine-Rich Protein 1, also known as FAM190A) has been reported as a fragile site for genomic rearrangement in cancers, with structural defects being reported in 40% of human cancers.^29^ More recently, knockdown of *CCSER1* was found to cause cell division defects and to interact with NDEL1, a dynein regulator that plays a role in neurodevelopment and adult neurons and may be contributing to the defective dynein-dependent axon transport noted in neurodegenerative disease like Huntington’s and PD.^30^ The human HERC gene family comprises 6 members, encoding 2 large and 4 small proteins, which have in common a HECT ubiquitin E3-ligase domain and an RLD domain.^31^ Localization of HERC3, *HERC5*, and *HERC6* (all small HERCs) to chromosome 4 indicates recent evolution and *HERC5* is the youngest member and primate-specific. Interestingly, a recent article shows that HERC5 can regulate activity of ubiquitin E3 ligase parkin by ISG15 conjugation.^32^

Our findings in this patient with an early-onset familial form of PD expand the non-motor clinical and neuropathological phenotype of *SNCA* triplication. The genetic array analysis of the *SNCA* triplication locus demonstrates the value of high-resolution CGH arrays for the detection of copy number variants, including discrimination of duplicated and triplicated regions and refined breakpoint mapping.

## Methods

### SNCA triplication cases

Genomic DNA was isolated from blood for our patient, from brain for the Lister case, and from a lymphoblastoid cell line from an additional Iowa kindred case (ND00139, NINDS Repository, Coriell Institute, Camden, NJ) for the purpose of fine-mapping the duplication and triplication breakpoints.

The study was approved by an Institutional Review Board and patients who participated in this study provided written informed consent.

### Clinical assessments

A complete medical history was obtained followed by a general medical and neurological examination on the Iowa kindred patient. Five minute resting ECG was taken to assess the heart rate variability. Unified Parkinson’s Disease Rating Scale, Hoehn and Yahr stage were noted and cognitive assessment was performed by using the Montreal Cognitive Assessment (MOCA). Color vision was tested by Farnsworth-Munsell 100 Hue Test (X-rite, Grand Rapids, MI, USA), 40-item University of Pennsylvania Smell Identification Test (UPSIT, Sensonics, Inc, Haddon Heights, HJ, USA) was taken to assess olfaction. Sleeping habits were assessed by questionnaires on REM sleep behavior disorder including Epworth Sleepiness Scale.

### Heart rate variability (HRV)

HRV was assessed by analyzing the normal R-R intervals of a five-minute supine waking EKG. The HRV parameters measured included the times domain parameters of standard deviation of R-R intervals (SDNN), and the percentage of consecutive RR intervals differing by more than 50 milliseconds (pNN50), the geometric parameter RR triangular index (RRTRI), the minor axis standard descriptor of the Poincaré plot (SD1), and the frequency domain parameters normalized Low Frequency (LF nu) and the ratio of LF/HF. While SDNN, RRTRI, and LF are thought to represent overall HRV influenced by both sympathetic and vagal systems, the parameters SD1 and pNN50 are considered parasympathetically dominated and LF/HF ratio indicates sympathovagal balance.

### Copy number variant (CNV) analysis

A comparative genomic hybridization (CGH) microarray (Design ID 021529, Agilent Technologies, Santa Clara, CA) comprising ~1 million (1 M) oligonucleotide probes uniformly distributed across the genome (2.1 Kb median probe spacing) was used to refine the breakpoints of the SNCA triplication cases. Genomic DNA samples were dye-labeled and hybridized to the 1 M CGH array according to the manufacturer’s instructions. Genomic DNA for all samples was labeled with Cy3 dye. To accurately assess CNVs across all chromosomes, sex-matched hybridizations were performed using a healthy male reference DNA sample (Population Bio, New York, NY), which was labeled with Cy5 dye. Array experiments were performed as a service by Oxford Gene Technology (Oxford, UK). The arrays were scanned using the Agilent microarray scanner, at 2 µm resolution (16 bit) and data was extracted using Feature extraction software version 10.7.3.1, grid design file 021529_D_F_20091001 and protocol CGH_107_Sep09. CNV analysis was performed using DNAcopy, a circular binary segmentation algorithm (from the R Bioconductor package), with log2ratio cutoffs of −0.35 for losses and +0.35 for gains. Array CGH data points were also manually inspected to verify the breakpoints called by the DNAcopy algorithm.

In order to further fine-map the *SNCA* breakpoints, we designed a high-density 8 × 60 K custom CGH array using the Agilent eArray web portal (Agilent Technologies, Santa Clara, CA, USA). In addition to Agilent control and normalization probes, the array consisted of 52,826 probes designed to interrogate 21 genes of interest, either in their entirety or for the purpose of fine mapping of previously identified breakpoints of interest in these genes, including *SNCA*. The median probe spacing was 24 bp for the left breakpoint and 46 bp for the right breakpoint. The Iowa kindred male (case report) and female (Coriell ND00139) genomic DNA samples were labeled and hybridized using the methods described for the 1 M CGH array, including sex-matched co-hybridization with a healthy male or healthy female reference DNA (Population Bio, New York, NY). Arrays were scanned, data extracted (grid design file IS-62976-8-V2_8by60K_cGH_Hs_20080925), and CNV analysis was performed using the same methods described for the 1 M CGH array.

### Histopathology

At the time of the autopsy the brain was divided in the sagittal plane, with the entire left hemibrain frozen at −70 °C and the right hemibrain fixed in 10% neutral buffered formalin. Multiple sections of neocortex, hippocampus, basal forebrain, basal ganglia, thalamus, midbrain, pons, medulla and cerebellum were embedded in paraffin and sections were examined with H&E microscopy. Sections of cortex, hippocampus, basal forebrain and brain stem were also stained with immunocytochemical methods and antibodies to alpha-synuclein (NACP98, Mayo Clinic, non-commercial^4,33^), phospho alpha-synuclein (pSyn#64, mouse monoclonal, Wako, Cat. No. 015-25191, dilution 1:10,000) and tau antibody CP13(tau phospho Ser202, mouse monoclonal, non-commercial, Peter Davies, Albert Einstein College of Medicine, Bronx, N.Y.^34^).

### iPSCs maintenance, propagation, and dopaminergic differentiation

iPSCs from our Iowa *SNCA* triplication patient and sibling control^35^ were cultured and maintained on Geltrex (Thermo Fisher, catalog # A1413302) in Essential 8 media (Thermo Fisher, catalog # A1517001). Cells were propagated every 7 to 8 days manually without enzymatic treatment.

iPSCs were differentiated using a commercially available dopaminergic (DA) neuron differentiation kit (Thermo Fisher, catalog # A3147701) according to manufacturer’s instructions. The yield of TH neurons was 25–35% and >90% total neurons (beta-III-tubulin, TUJ1) from iPSCs after 35 days in vitro.

### mRNA expression analysis

Total mRNA was isolated from iPSCs and neurons with RNeasy Micro Kit (Qiagen, catalog # 74004), following the manufacturer’s instructions. Two micrograms were used to synthesize cDNA (20 μl per reaction volume) using the iScript™ cDNA Synthesis Kit (Bio-Rad, catalog # 170-8890) in a Bio-Rad DNAEngine Peltier thermal cycler. Quantitative PCR analyzation of 1 μl of 10 ng/ul cDNA was performed in triplicates on a CFX96 Real time system Thermal cycler (Bio Rad). The primers/probes used for real-time amplification for HERC3 was FAM-MGB labeled HERC3 (Thermo Fisher, Assay ID: Hs01040150_m1), for HERC5 was FAM-MGB labeled HERC5 (Thermo Fisher, Assay ID: Hs00180943_m1), for HERC6 was FAM-MGB labeled HERC6 (Thermo Fisher, Assay ID: Hs00215555_m1), for aSyn was FAM-MGB labeled SNCA (Thermo Fisher, Assay ID: Hs00240906_m1), for MMRN1 was FAM-MGB labeled MMRN1 (Thermo Fisher, Assay ID: Hs01113299_m1), for CCSER1 was FAM-MGB labeled CCSER1 (Thermo Fisher, Assay ID: Hs00286784_m1), for TH was FAM-MGB labeled TH (Thermo Fisher, Assay ID: Hs00165941_m1), and for normalization VIC-MGB_PL labeled GAPDH (Thermo Fisher, catalog # 4326317E). Relative expression levels were calculated with subsequent ΔCT values that were analyzed using CFX software. Comparative ΔΔCT method was used to normalize with subsequent CT values to the housekeeping gene GAPDH.

### Data availability

The data generated during and/or analyzed during the current study are available from the corresponding author on reasonable request.

## Electronic supplementary material

Supplemental data
