# Supplementary material for: Genetic fine-mapping of the Iowan SNCA gene triplication in a patient with Parkinson’s disease
Source: NPJ Parkinsons Dis. 2018 Jun 15;4:18. doi: 10.1038/s41531-018-0054-4 (PMC6003950; doi:10.1038/s41531-018-0054-4)
Supplement: Supplementary file 1 — Supplemental data [file 41531_2018_54_MOESM1_ESM.pdf]

Supplemental Data.

Genetic fine-mapping of the Iowan *SNCA* gene triplication in a patient with Parkinson's disease

Faria Zafar[a], Ruksana Azhu Valappil[a], Sam Kim[a], Krisztina K. Johansen[b], Anne Lynn S. Chang[c], James W. Tetrud[a], Peggy S. Eis[e], Eli Hatchwell[e], J. William Langston[a] , Dennis W. Dickson[f], Birgitt Schüle[a*]

[a]Parkinson's Institute and Clinical Center, Sunnyvale, CA, U.S.A.

[b]Department of Neurology, Akershus University Hospital, Lorenskog, Norway

[c]Department of Dermatology, Stanford University School of Medicine, Stanford, CA, U.S.A.

[d]Stanford Neuroscience Health Center, Stanford School of Medicine, Stanford, CA, U.S.A

[e]Population Bio, Inc., New York, NY, U.S.A.

[f]Neuropathology Laboratory, Mayo Clinic, Jacksonville, FL, U.S.A.

Supplemental Table 1. Lewy body count in cortical regions in *SNCA* triplication patient

**Cortical Region**	**Lewy bodies (per 20x field)**
Mid-frontal	5-7
Superior temporal	10-15
Inferior parietal	3-5
Cingulate gyrus	12-15
Parahippocampal gyrus	15-18

Supplemental Table 2: *SNCA* chromosomal duplication/triplication breakpoints (chr4, GRCh37/hg19)*

	Lister Male			Iowa Male (case report)			Iowa Male (case report)			Iowa Female		
	1M aCGH				1M aCGH			Custom aCGH			Custom aCGH	
Copy number	Start	Stop	Size	Start	Stop	Size	Start	Stop	Size	Start	Stop	Size
2	n/a	90318074		n/a	89320408		n/a	89322949		n/a	89322949	
3	unknown	unknown		89324508	89336598	12090	89322975	89337274	14299	89322975	89337274	14299
4	90323528	91140682	817154	89338268	91047347	1709079	89337388	91047347	1709959	89337388	91047347	1709959
3	unknown	unknown		91050731	91054535	3804	91047421	91059278	11857	91047421	91059278	11857
2	91142684	n/a		91060085	n/a		91059510	n/a		91059510	n/a	

* The Lister Male was not run on the custom CGH array and the Iowa Female was not run on the 1M CGH array.

Supplemental Figure 1

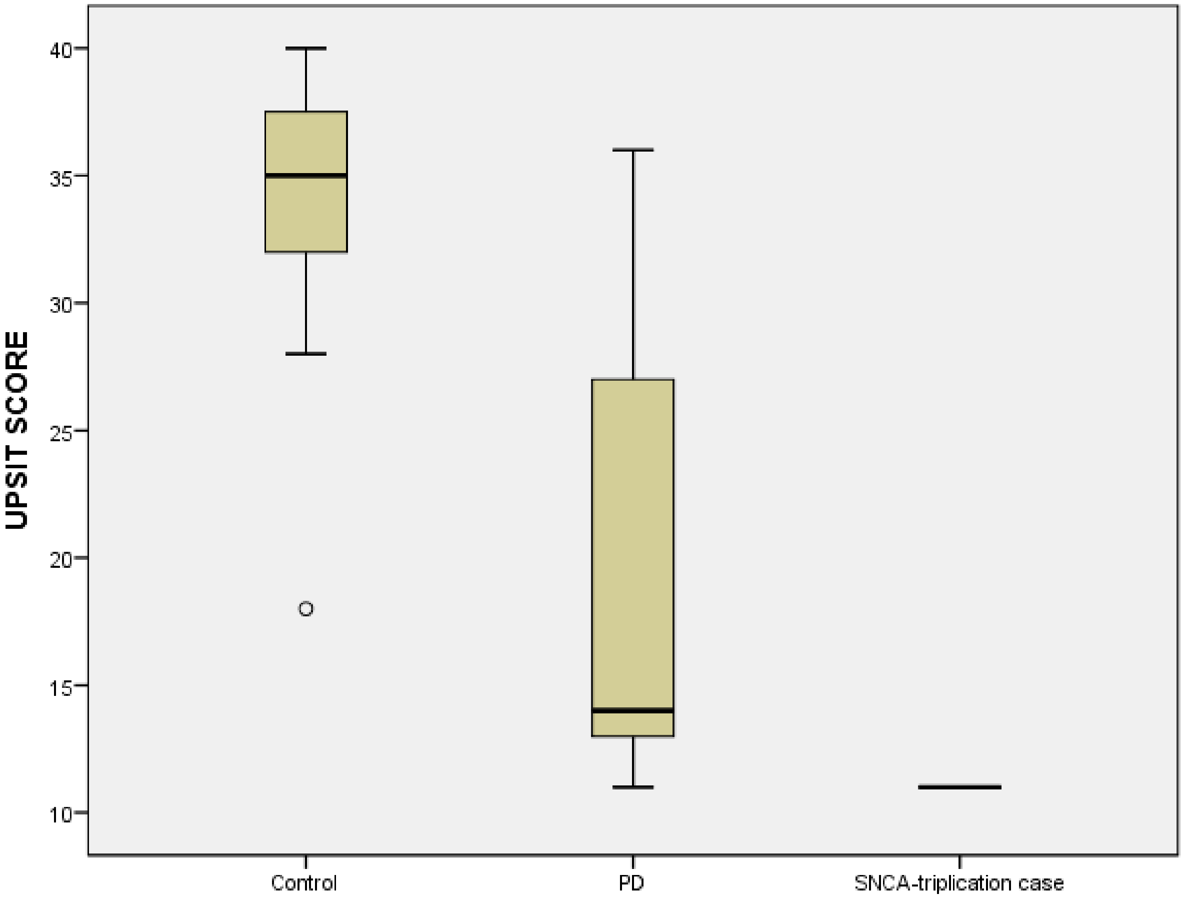

Supplemental Figure 1 legend. Reduced sense of smell observed in the Iowa kindred SNCA triplication patient. UPSIT scores of controls (n=11, 64.7 ± 6.9, age range 55 to 76 years), PD (n=9, 70.4 ± 10.0 years, age range 56 to 82 years) and SNCA triplication patient (45 years). The SNCA triplication patient has the lowest UPSIT score of 11. For his age group, a score of 27/40 would rank in the 5th percentile.

Supplemental Figure 2

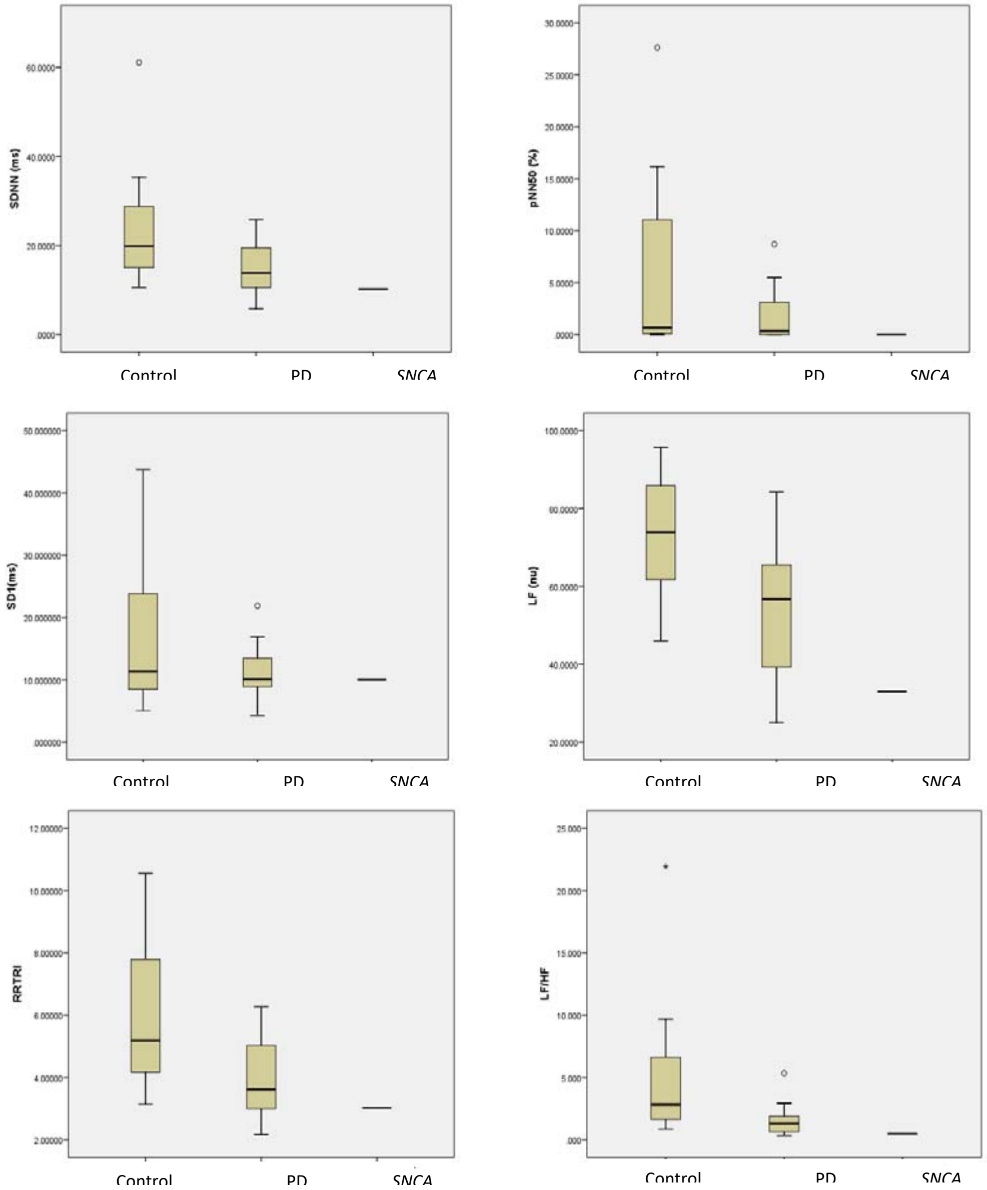

Supplemental Figure 2 legend. Reduced Heart Rate Variability (HRV) observed in the Iowa kindred SNCA triplication patient. Patients diagnosed with idiopathic PD by a movement disorder specialist were enrolled from the clinic at Parkinson's Institute (n=11; 70.8 ± 9.0 years, age range 56 to 82 years, Hoehn & Yahr 1-3, 6 men and 5 women, all white). Control subjects were age matched volunteers (n=11; 64.7 ± 6.9 years, age range 55 to 76 years, 8 men and 3 women, all white), SNCA triplication patient (45 year old male). The HRV parameters shown are SDNN, pNN50, RRTRI, SD1, LF normalized and LF/HF ratio. The SNCA triplication patient has lower HRV than idiopathic PD patients. All methods of measurement of HRV have been previously described in detail [7].

Supplemental Figure 3

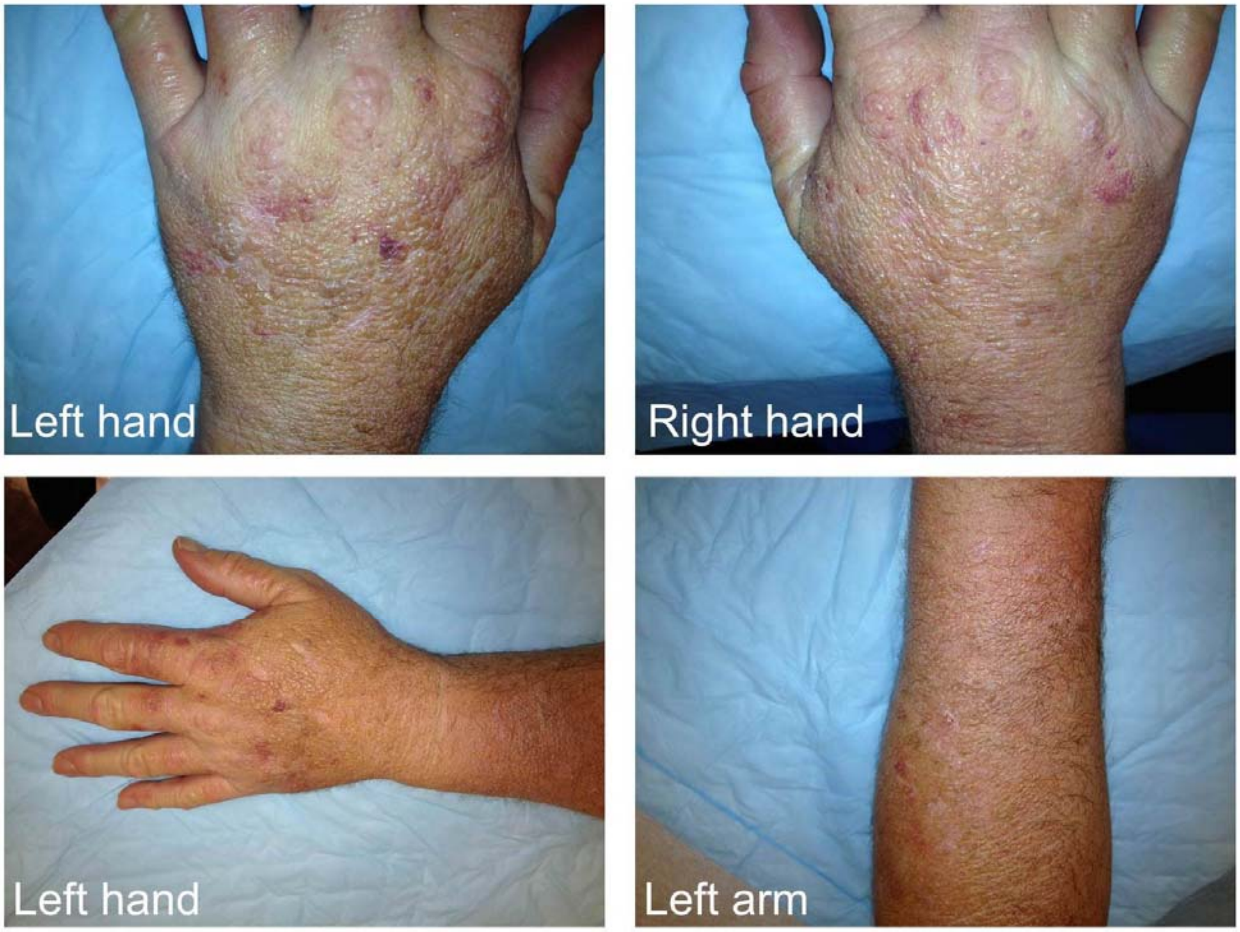

Supplemental Figure 3 legend. Colloid milium skin pictures for Iowa kindred SNCA triplication patient. Skin lesions are symmetrical on left and right dorsum of the hands and on sun-exposed areas of the lower arms. Lesions appear as small firm yellow nodules.

Supplemental Figure 4

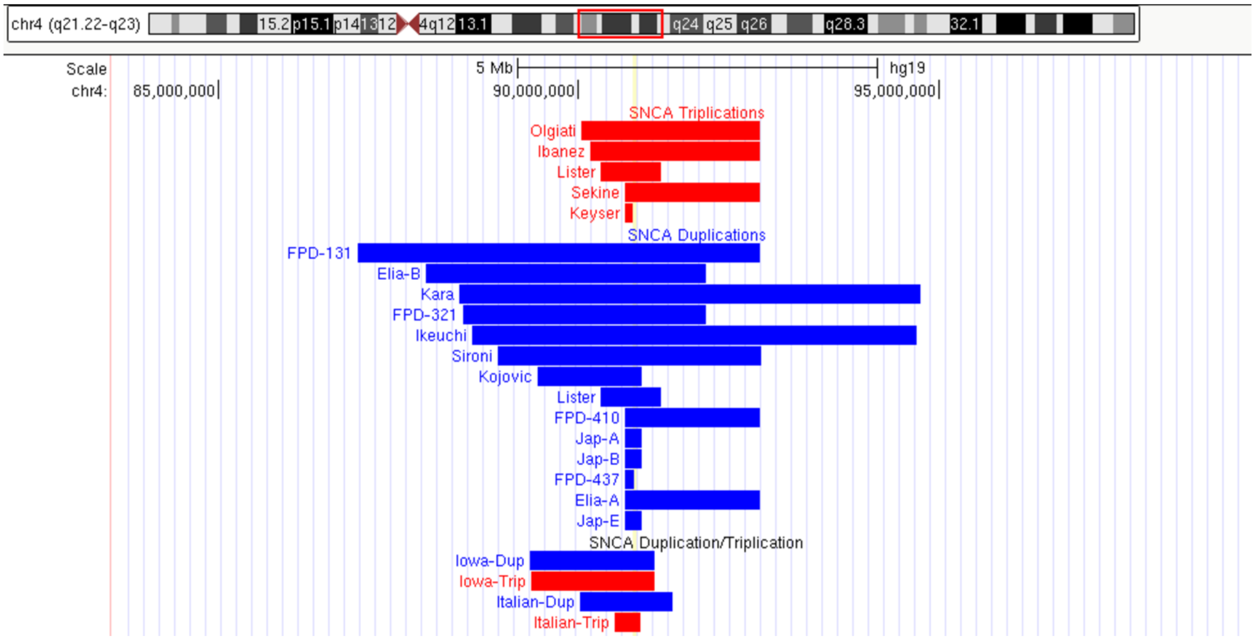

Figure Legend Supplemental Figure 4. Families with *SNCA* duplication/triplication copy number variants. UCSC genome browser custom tracks for genomic locus chromosome 4q21.23-q22.3 (GRCh37/hg19, chr4:84,239,011-98,739,011) and chromosome 4q22.1 (GRCh37/hg19, chr4:90,608,984-90,793,984). CNVs of *SNCA* locus; colors indicate gene copy numbers red: SNCA CNV triplications; blue: *SNCA* CNV duplications; combination: *SNCA* triplication (red) and duplication (blue); green. IDs given to tracks were either based on family ID from literature or are first authors name of publication the case/family has been reported. Of note, this supplemental figure is modified from Figure 1A in Piper et al. 2018[8].

Supplemental Figure 5

SNCA left genomic breakpoint

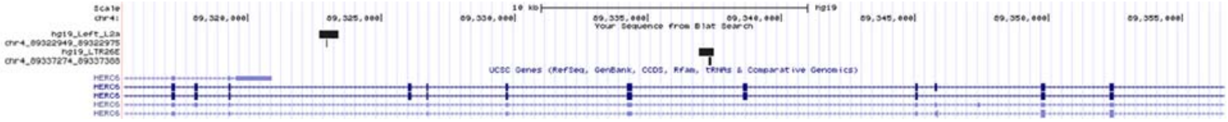

SNCA right genomic breakpoint

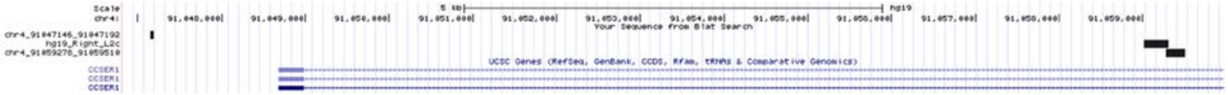

Figure Legend Supplemental Figure 5: Analysis of *SNCA* genomic triplication breakpoint regions (GRCh37/hg19). *SNCA* left genomic breakpoint: According to the custom array, the left wildtype-duplication breakpoint lies between chr4:89322949 and chr4:89322975. This is a distance of only 26bp. There is a LINE repeat at that position, which is L2a (member of L2 family). The left duplication-triplication breakpoint lies between chr4:89337274 and chr4:89337388. This is a distance of only 114bp. There is an LTR repeat at that position, which is LTR26E (member of ERV1 family).

***SNCA* right genomic breakpoint**: According to the custom array, the triplication-duplication breakpoint lies between chr4:91047146 and chr4:91047192. This is a distance of only 46bp. There is no repeat annotated at this position. The right duplication-wildtype breakpoint lies between chr4:91059278 and chr4:91059510. This is a distance of only 232bp. There is a LINE repeat at that position, which is L2c (member of L2 family). Of note, L2a and L2c elements might no overt homology.

Supplemental Figure 6

A

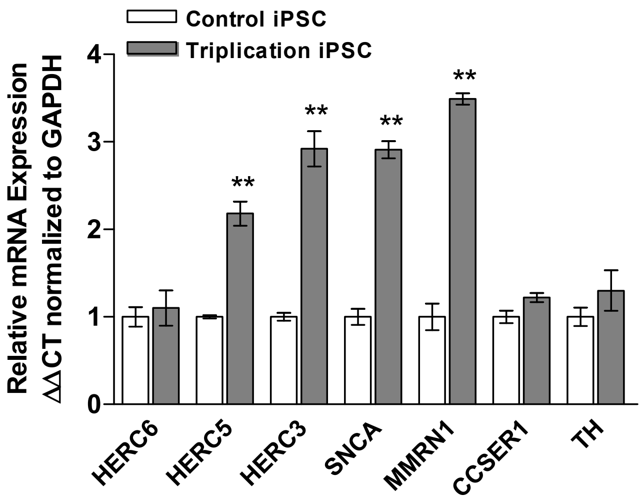

B

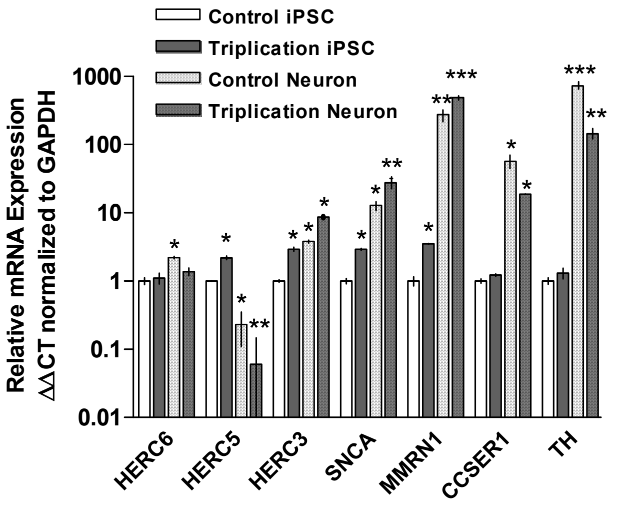

Figure Legend Supplement Figure 6. Expression analysis of genes at the SNCA triplication breakpoint regions in iPSCs and in mature neurons. mRNA expression of gene of interest are quantitatively measured using taqman probe assay. A) fold change in mRNA expression of SNCA triplication iPSCs compared to control iPSCs. B) fold change in mRNA expression of SNCA triplication iPSCs, control neurons, and SNCA triplication neurons compared to control iPSCs. Assay is normalized to the housekeeping gene, GAPDH. Data represents one trial from three independent experiments. Error bars represent the mean and standard error from triplicates per experiments. $^*p \leq 0.05$, $^{**}p \leq 0.01$, and $^{***}p \leq 0.001$.

References

1 Singleton, A. B. *et al.* alpha-Synuclein locus triplication causes Parkinson's disease. *Science* 302, 841 (2003).

2 Farrer, M. *et al.* Comparison of kindreds with parkinsonism and alpha-synuclein genomic multiplications. *Annals of neurology* 55, 174-179 (2004).

3 Beach, T. G. *et al.* Evaluation of alpha-synuclein immunohistochemical methods used by invited experts. *Acta neuropathologica* 116, 277-288, doi:10.1007/s00401-008-0409-8 (2008).

4 Gwinn-Hardy, K. *et al.* Distinctive neuropathology revealed by alpha-synuclein antibodies in hereditary parkinsonism and dementia linked to chromosome 4p. *Acta neuropathologica* 99, 663-672 (2000).

5 Herskovits, A. Z. & Davies, P. The regulation of tau phosphorylation by PCTAIRE 3: implications for the pathogenesis of Alzheimer's disease. *Neurobiology of disease* 23, 398-408, doi:10.1016/j.nbd.2006.04.004 (2006).

6 Byers, B. *et al.* SNCA triplication Parkinson's patient's iPSC-derived DA neurons accumulate alpha-synuclein and are susceptible to oxidative stress. *PloS one* 6, e26159, doi:10.1371/journal.pone.0026159 (2011).

7 Valappil, R. A. *et al.* Exploring the electrocardiogram as a potential tool to screen for premotor Parkinson's disease. *Movement disorders : official journal of the Movement Disorder Society* 25, 2296-2303, doi:10.1002/mds.23348 (2010).

8 Piper, D. A., Sastre, D. & Schüle, B. Advancing Stem Cell Models of Alpha-Synuclein Gene Regulation in Neurodegenerative Disease. *Frontiers in neuroscience* 12, doi:10.3389/fnins.2018.00199 (2018).
